# Open questions: A logic (or lack thereof) of genome organization

**DOI:** 10.1186/1741-7007-11-58

**Published:** 2013-05-28

**Authors:** Laurence D Hurst

**Affiliations:** 1 Department of Biology and Biochemistry, University of Bath, Bath BA2 7AY, UK

## 

As a graduate student I was advised that if you don’t understand why an animal does what at first sight looks like behavior contrary to its best interests, then you should presume that it is you, not the animal, that is stupid. Look harder, the wisdom goes, and you will discover natural selection’s cunning logic. While this may be good advice to those studying organismic behavior or anatomy, when we approach genomic anatomy and behavior it will not do.

Indeed, typically when thinking about genomes people often make the opposite presumption. Intergenic DNA was dismissed as irrelevant junk and many transcripts are presumed to be just so much noise. Synonymous mutations have been assumed to be neutrally evolving and where in a genome a gene sits is considered to be largely irrelevant. But are these assumptions more witness to a lack of understanding rather than robust statements about how genomes function and evolve? You are, after all, alive reading this, testament to the fact that your genome is doing something right.

So then, what features of our and other genomes are functionally relevant and which just so much noise? More importantly, when selection does act, why is it acting?

The challenge is difficult. Assuming that sites involved in interactions are all functional isn’t good enough. By this, the logic employed by ENCODE [1], following a collision between a car and a pedestrian, a car’s bonnet would be ascribed the 'function' of projecting a pedestrian many meters and the pedestrian would have the 'function' of deforming the car’s bonnet. Similarly, we expect, for example, accidental transcription factor-DNA binding to go on at some rate, so assuming that transcription equals function is not good enough. The null hypothesis after all is that most transcription is spurious and alternative transcripts are a consequence of error-prone splicing. Conversely, assuming unbound sequence, such as nucleosome-free regions, to be lacking in function can mislead, as they can be critical for the proper control of gene expression [2].

Many approaches to the question have looked for statistical signatures of sequence under selective constraint. However, selection could, for example, be on the process of transcription not the product of transcription. A stronger, or perhaps complementary, approach is to start with a mechanistic hypothesis. If you know splice sites need exonic splice enhancer motifs to define them, then do these motifs impact on the evolution of the protein and gene sequence within which they are embedded [3]? As nucleosome location is important for gene expression, then does selection act on the DNA level to maintain proper positioning? Does this mean that a single point mutation can be disfavored for a minor disruption of function? We know that genes close together on chromosomes tend to be expressed together [4,5]. Are genome rearrangements favored or disfavored then for bringing combinations together or breaking them up? How often will selection care about single point mutations within microRNA pairing sites? Do genes evolve to avoid pairing with certain microRNAs [6]? The list of questions goes on (and should keep us in business for a while yet).

The questions are not simply of academic interest. If you know where and why selection is acting to maintain the status quo, you know better which mutations will be disease-causing mutations and why. You also might be better able to manage the risk of introducing genes into genomes. Early gene therapy trials were, for example, halted owing to unforeseen knock-on consequences of gene insertion [7].
